# Pyrazinamide-resistant Tuberculosis Obscured From Common Targeted Molecular Diagnostics

**DOI:** 10.1016/j.drup.2023.100959

**Published:** 2023-04-06

**Authors:** Samuel J. Modlin, Mikael Mansjö, Jim Werngren, Chidera M. Ejike, Sven E. Hoffner, Faramarz Valafar

**Affiliations:** a Laboratory for Pathogenesis of Clinical Drug Resistance and Persistence, School of Public Health, San Diego State University, San Diego, CA, USA; b Department of Microbiology, Public Health Agency of Sweden, Solna, Sweden; c Department of Global Public Health, Karolinska Institute, Stockholm, Sweden

**Keywords:** Pyrazinamide, Heteroresistance, Molecular diagnostics, Tuberculosis

## Abstract

Here, we describe a clinical case of pyrazinamide-resistant (PZA-R) tuberculosis (TB) reported as PZA-susceptible (PZA-S) by common molecular diagnostics. Phenotypic susceptibility testing (pDST) indicated PZA-R TB. Targeted Sanger sequencing reported wild-type PncA, indicating PZA-S TB. Whole Genome Sequencing (WGS) by PacBio and IonTorrent both detected deletion of a large portion of *pncA*, indicating PZA-R. Importantly, both WGS methods showed deletion of part of the primer region targeted by Sanger sequencing. Repeating Sanger sequencing from a culture in presence of PZA returned no result, revealing that 1) two minority susceptible subpopulations had vanished, 2) the PZA-R majority subpopulation harboring the *pncA* deletion could not be amplified by Sanger primers, and was thus obscured by amplification process. This case demonstrates how a small susceptible subpopulation can entirely obscure majority resistant populations from targeted molecular diagnostics and falsely imply homogenous susceptibility, leading to incorrect diagnosis. To our knowledge, this is the first report of a minority susceptible subpopulation masking a majority resistant population, causing targeted molecular diagnostics to call false susceptibility. The consequence of such genomic events is not limited to PZA. This phenomenon can impact molecular diagnostics’ sensitivity whenever the resistance-conferring mutation is not fully within primer-targeted regions. This can be caused by structural changes of genomic context with phenotypic consequence as we report here, or by uncommon mechanisms of resistance. Such false susceptibility calls promote suboptimal treatment and spread of strains that challenge targeted molecular diagnostics. This motivates development of molecular diagnostics unreliant on primer conservation, and impels frequent WGS surveillance for variants that evade prevailing molecular diagnostics.

Pyrazinamide (PZA) is an important drug in the treatment of tuberculosis (TB). PZA resistance is acquired by *M. tuberculosis* primarily through mutations in the *pncA* gene that diminish the expression of pyrazinamidase/nicotinamidase (PZase) (Lamont et al., 2020) or reduce its enzymatic activity (Junaid et al., 2019; Khan et al., 2019), thereby reducing PZase-mediated conversion of PZA to its active form, pyrazinoic acid (POA). Numerous unique *pncA* mutations reportedly confer resistance through this mechanism (Yadon et al., 2017; Ramirez-Busby and Valafar, 2015). Missense mutations in *pncA* are the most common PZase-inactivating mutations, but small deletions (Yadon et al., 2017), promoter mutations (Ramirez-Busby and Valafar, 2015; Sheen et al., 2013), and larger deletions (Aono et al., 2014) have been reported as well.

Recently, we identified a clinical isolate with a perplexing case of PZA-monoresistance (Modlin et al., 2021). Here, we sought to characterize the nature of this discordance and determine how it would affect the accuracy of common and emerging targeted molecular diagnostic methods. This isolate was genotyped as majority *pncA*_*WT*_ with a minor population of *pncA*_*Ser65Ser*_ (*pncA*_*WT*_/*pncA*_*Ser65Ser*_) by targeted Sanger sequencing (both of which are PZA-S) but genotyped by Whole Genome Sequencing (WGS) with PacBio SMRT-sequencing as a single population with a large deletion spanning the first 158 nucleotides of *pncA* and 264 bp upstream of its start (*pncA*_*del-264:158*_). We repeated targeted Sanger sequencing following growth in PZA MGIT tube, but the *pncA* primers did not return anything to sequence. Observing this, we next sequenced the drug-containing MGIT-derived sample on IonTorrent (Werngren et al., 2017), which, like SMRT-sequencing, exclusively recovered *pncA*_*del-264:158*_ (Table 1). The evidence to this point left the nature of these discrepancies unclear. The *pncA*_*WT*_/*pncA*_*Ser65Ser*_ mixture recovered by the initial Sanger assay (Supplemental Fig. 1) suggested mixed infection. Cross-contamination in the Sanger assay is highly unlikely considering that none of the samples in the originally tested plate had the *pncA*_Ser65Ser_ variant, and the presence of both PZA-S subpopulations in the Sanger Sequencing performed by a third party (Supplemental text).

Taking all evidence (Table 1) together, we conclude that the *pncA*_WT_/*pncA*_Ser65Ser_ proportion in the sample was too small to appear in IonTorrent sequencing (Fig. 1). Given the sequencing depth (initial IonTorrent culture) at the remaining portion of *pncA* was ~50x, this is quite plausible. The numerous (35) PCR cycles preceding Sanger sequencing amplify *pncA*_WT_ and *pncA*_Ser65Ser_ but do not amplify the subpopulations with the *pncA* deletion (as the primer region is absent), thereby massively increasing the fractional abundance of *pncA*_*WT*_ and *pncA*_Ser65Ser_ relative to *pncA*_*del-264:158*_. This creates a curious and clinically important case where a resistance-conferring majority subpopulation is missed due to primer evasion.

After elucidating that the majority PZA-R subpopulation (*pncA*_*del-264:158*_) with PZA-S subpopulations (*pncA*_WT_/*pncA*_Ser65Ser_) accounted for the discrepant DST results, we investigated whether existing primer schemes and emerging primer-based amplification methods (Banu et al., 2017; Hameed et al., 2020; Willby et al., 2017; Jouet et al., 2021; Kahbazi et al., 2018; Tam et al., 2019; Li et al., 2021; Liu et al., 2023; Mansjo et al., 2017; Rowneki et al., 2020; Sengstake et al., 2017; Tan et al., 2014) would capture the resistance-conferring *pncA*_*del-264:158*_. None of the *pncA* amplification schemes had primers spanning the deletion boundaries of this majority PZA-R subpopulation (Fig. 2); because of this, all would falsely suggest a drug-susceptible infection. Thus, current molecular PZA susceptibility diagnostics (targeted Sanger sequencing), a Line Probe Assay (LPA) recently endorsed by the WHO (World Health Organization, 2021) for PZA gDST of Multidrug-Resistant tuberculosis (MDR-TB) (NiPro Genoscholar^™^ PZA-TB II (Willby et al., 2017)), and those proposed for targeted NGS PZA molecular diagnostics (Mohamed et al., 2021) all share this vulnerability (Fig. 2). This blind spot would remain for any *pncA* deletion of their primer sequence accompanied by minority PZA-S subpopulations.

Targeted NGS approaches under development for TB molecular resistance diagnostics, such as deeplex (Jouet et al., 2021), have garnered recent excitement due to their potential to rapidly query larger swaths of resistance genes than LPAs or other nucleic acid amplification tests (Mohamed et al., 2021). Our findings suggest that expanding the region flanking resistance-conferring genes may be prudent, as the narrow flanking region targeted by deeplex would introduce the same blindness to the majority PZA-R population we report here for targeted PZA gDST (Fig. 2). The targeted Sanger primers for this study extended 196 nucleotides upstream and 175 downstream of the *pncA* start (Juréen et al., 2008), the upstream portion of which was ablated by the *pncA* deletion (Fig. 2). Extending primer sites further down the flanking sequence of the resistance marker could reduce the incidence of this kind of resistance misclassification, yet it remains unclear how large of deletions need be accommodated during primer design. Additional challenges to widespread implementation of targeted assays that extend further around the target gene include the cost of changing infrastructure and training staff. One could argue that these atypical cases are too rare to justify changes in PZA gDST practices. However, genotyping studies typically lack sequencing methods that would catch such instances. Therefore, prevalence of variants that evade targeted molecular diagnostics may be underestimated systematically.

Even if one assumes such instances are uncommon, outbreak of a strain harboring this type of resistance could cause mistreatment of many patients and further spread before proper characterization. Concerningly, uncommon resistance mechanisms are increasing in prevalence for first-line anti-TB drug isoniazid and evasion of molecular diagnostics appears to be the most common source driving this trend (Valafar, 2021). Without prospective surveillance for such events, broad reliance on targeted molecular diagnostics will artificially select for strains that evade detection by commonly used diagnostics. Resistance conferred by loss-of-function mutations in non-essential genes incur negligible fitness cost, ostensibly making them more tolerant to large deletions or insertions and thus more prone to develop resistance in this manner. This threat of undetected resistance moving through a population is especially prudent to consider for initiatives proposing programmatic replacement of phenotypic DST (pDST) with gDST. Purely gDST approaches that implement untargeted WGS—like that recently implemented for TB DST in the United Kingdom (Park et al., 2022)—would capture the resistance appropriately. Others, meanwhile, have argued for targeted diagnostics for gDST to decrease turnaround time (Cabibbe et al., 2020). Undoubtedly, turnaround time is an important consideration, but our findings impel parallel WGS to ensure cases of false susceptibility do not slip through and disseminate unabated through the population. While non-targeted short-read WGS could capture some cases of primer-evading deletions like we observed here, insertion sequences sometimes cause resistance to TB drugs, including PZA-R from IS6110 insertions into *pncA* (Antoine et al., 2021), making use of long-read technologies prudent. Accordingly, in settings considering programmatic use of targeted gDST methods, we advocate for initiatives that implement long-read sequencing of susceptible isolates to prospectively surveil for primer-evading resistant variants. Such initiatives would mitigate the public health risk of TB with cryptic DR spreading.

Amplification-based diagnostics implicitly assume all subpopulations are amplified equivalently. This assumption is crucial if ruling out resistance via targeted molecular diagnostics. We have highlighted an important violation of this assumption: a large deletion that confers resistance while evading the primers designed to readout resistance status. This risk of targeted approaches identifying small susceptible subpopulations while obscuring the predominant, resistant population in the sample applies to molecular resistance diagnostics for other diseases as well. In any case where deletions span the primer-targeted region, minority subpopulations could cause erroneous susceptibility calls, even when they are comparatively small—even when smaller than the limit of detection for heteroresistance.

## Supplementary Material

Supplementary Material

## Figures and Tables

**Fig. 1.**
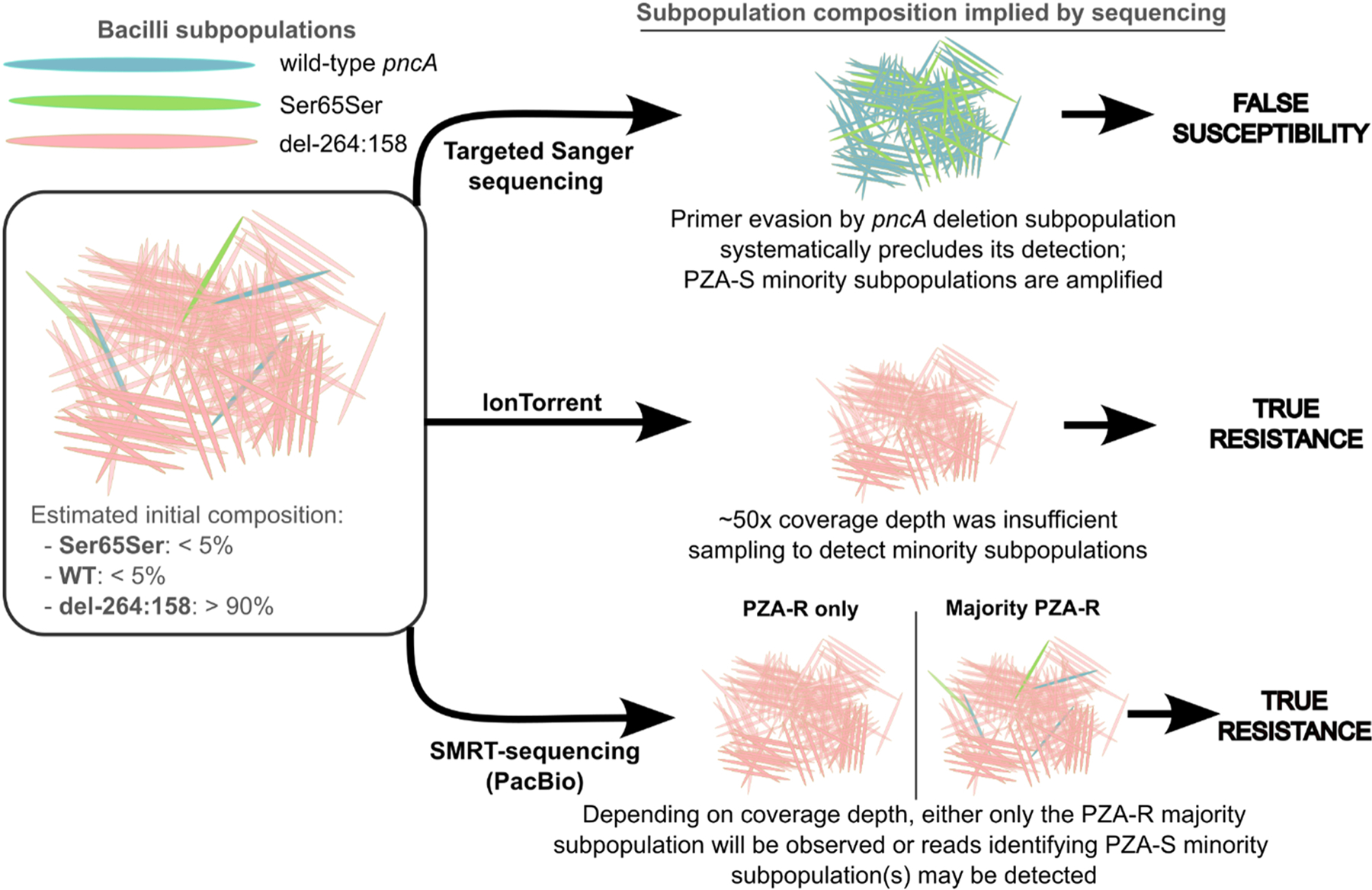
Model for initial composition of subpopulations that give rise to the gDST and pDST observed in this study. The fraction of each subpopulation we estimate was present in the original sample (left) contrasted with the composition of the three subpopulations’ DNA that would ultimately be sequenced (right) given the original composition.

**Fig. 2.**
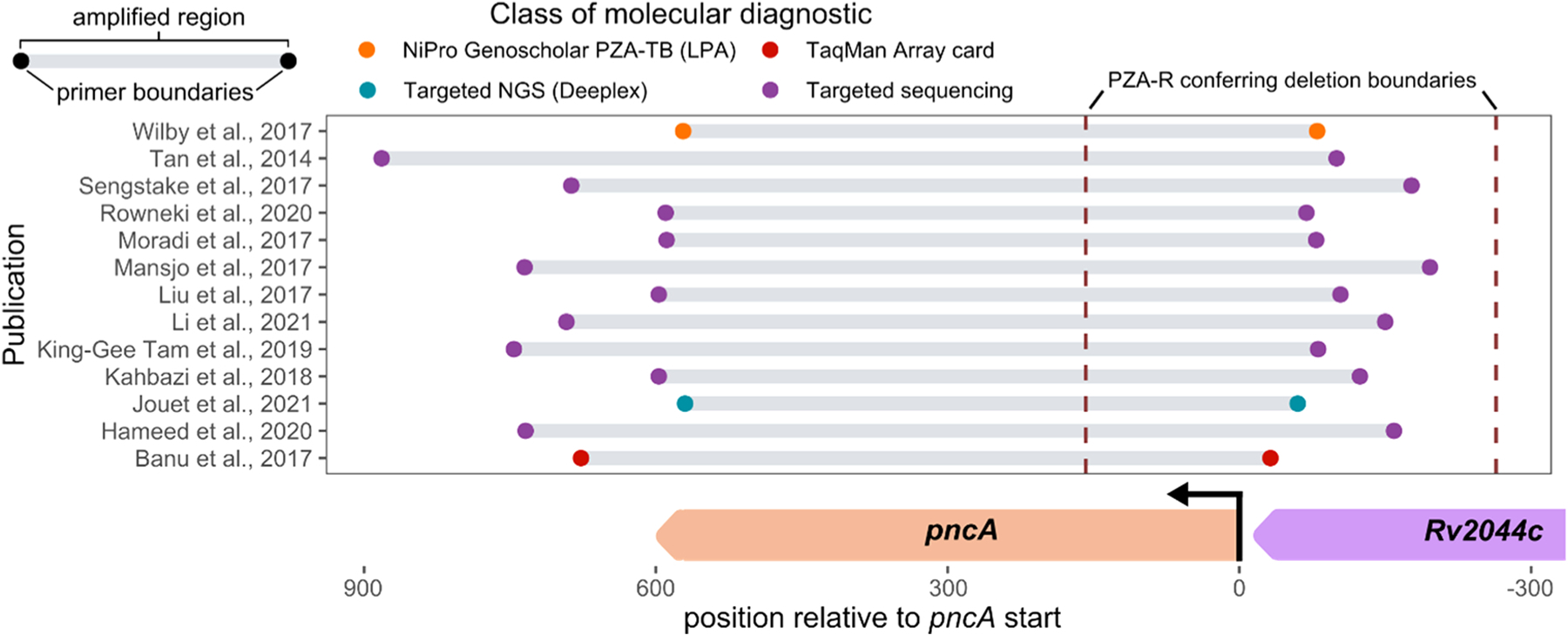
Undetected pyrazinamide resistance (PZA-R) conferring deletion with respect to primer schemes for targeted PZA-R molecular diagnostics. Primer sequence boundaries from previously published studies using PCR schemes for molecular investigation of *pncA* genotype. Dots indicate the inner boundary of primers in each scheme, which need to both be outside of the deleted region for the intervening sequences to be amplified and subsequently sequenced to identify the presence of mutations that confer PZA resistance. Location of the resistance conferring deletion and primer sequence boundaries are shown with respect to *pncA*.

**Table 1:** Outcomes of all genotypic (gDST) and phenotypic drug susceptibility tests (pDST) performed on the isolate of interest as part of this study.

Test	Concentration/media	Result
Bactec/MGIT	100 mg	Resistant
Bactec/MGIT	200 mg	Resistant
Sanger	Drug-free	*pncA*_*WT*_ and *pncA*_*Ser65Ser*_
Sanger	MGIT PZA tube	Primer fail
Sanger	Drug-free; 3rd party	*pncA*_*WT*_ and *pncA*_*Ser65Ser*_
Ion Torrent	Drug-free	*pncA*_*del-264:158*_
Ion Torrent	MGIT PZA tube	*pncA*_*del-264:158*_
PacBio WGS	Drug-free	*pncA*_*del-264:158*_
