# Supplementary material for: Pyrazinamide-resistant Tuberculosis Obscured From Common Targeted Molecular Diagnostics
Source: Drug Resist Updat. Author manuscript; Available in PMC 2023 Jul 3. (PMC10317212; doi:10.1016/j.drup.2023.100959)
Supplement: Supplementary Material [file NIHMS1907051-supplement-Supplementary_Material.zip › 1-s2.0-S1368764623000420-mmc1.docx]

**Supplemental Text**

**Cross-contamination highly unlikely explanation for mixed infection**

The *pncA_WT_*/*pncA_Ser65Ser_*  mixture recovered by the initial Sanger assay (Supplemental Figure 1) suggested mixed infection; *pncA*_Ser65Ser_ is a phylogenetic marker for some lineage 3 sublineages^8^ (CAS strains in subclades 3.1.1, 3.1.2) while initial spoligotyping indicated that the isolate belonged to H3 (SIT 50). Meanwhile, lineage-typing of both PacBio WGS in drug-free culture and Ion Torrent WGS of DNA extracted from the growth from in PZA MGIT tube indicated lineage 4.1.2.1 (Euro-American (Haarlem) "T1,H1"). Re-sequencing DNA from the initial culture with IonTorrent corroborated both the lineage 4.1.2.1 membership and harboring the *pncA_del-264:158_* deletion with no detectable level of *pncA*_WT_ or *pncA*_Ser65Ser_. Cross-contamination in the Sanger assay is highly unlikely considering that (1) none of the samples in the plate had the *pncA*_Ser65Ser_ variant; and (2) the targeted Sanger Sequencing run by a third-party laboratory (Genoscreen) also recovered the same two PZA-S subpopulations (*pncA_WT_* and *pncA_Ser65Ser_* ).

**Materials and Methods**

**Culture and DNA extraction**

*M. tuberculosis* samples were prepared and extracted at the World Health Organization Supranational Reference Laboratory (WHO SRL) in Stockholm, Sweden. Isolates growing on Löwenstein-Jensen medium were received from clinical TB laboratories in Sweden and subsequently Sanger sequenced. Sample preparation and DNA extraction were performed as described previously^6^.

**Pacbio SMRT-sequencing**

Extracted DNA was shipped from the reference laboratory in Stockholm and sequencing was performed at the Institute for Genomic Medicine at the University of California, San Diego. DNA libraries for PacBio (Pacific Biosciences, Menlo Park, CA) were prepared using PacBio’s DNA Template Prep Kit with no follow-up PCR amplification. Briefly, sheared DNA was end repaired, and hairpin adapters were ligated using T4 DNA ligase. Incompletely formed SMRTbell templates were degraded with a combination of Exonuclease III and Exonuclease VII. The resulting DNA templates were purified using SPRI magnetic beads (AMPure, Agencourt Bioscience, Beverly, MA) and annealed to a two-fold molar excess of a sequencing primer that is specifically bound to the single-stranded loop region of the hairpin adapters. SMRTbell templates were subjected to standard SMRT sequencing using an engineered phi29 DNA polymerase on the PacBio RS II system according to manufacturer's protocol.

**Sanger and IonTorrent sequencing**

Sanger sequencing of *pncA* was performed on the initial sample and repeated on the growth from the PZA MGIT tube. Observing the lack of Sanger sequencing data from the PZA MGIT tube, IonTorrent sequencing was carried out on DNA extracted from the PZA MGIT tube. DNA sequencing with Sanger and IonTorrent were performed at the WHO SRL in Stockholm, Sweden using previously described methods^6^. Confirmatory Sanger sequencing was performed by GenoScreen (Lille, France).

**Genotyping from SMRT-sequencing data**

Raw Pacific Biosciences SMRT-sequencing reads were aligned to the genome of *M. tuberculosis* virulent type strain H37Rv reference strain (Genbank: NC_000962.3) using BLASR^7^ (v1.3) with default parameters. PBHoover^8^ (<https://gitlab.com/LPCDRP/pbhoover>) corrected aligned reads and called variants based on a maximum likelihood criterion. VCF formatted files were further annotated with Variant Effect Predictor^9^ (VEP) (v87) to determine the consequence of each variant. Variants within and proximally flanking *pncA* were screened for using a custom python script (<https://gitlab.com/LPCDRP/drug-resistance/-/blob/master/src/known-resistance-association.py>). Lineage typing of subpopulations from the Pacific Biosciences’ long reads was performed using fast-lineage^10^

**Drug susceptibility testing**

PZA BACTEC MGIT 960 (Becton & Dickinson) was performed according to manufacturer instructions. The DST inoculum was prepared from bacterial growth on Löwenstein-Jensen egg medium in 37°C. Briefly, two 1 μl loops of bacteria were suspended in 3 ml phosphate buffered saline (PBS) in a small glass tube with glass beads. The bacterial suspension was homogenised using a vortex or an ultrasound water bath to disperse any clumps. The suspension was then left to sediment for 20 min and the upper 2 ml were transferred to a new tube and left to sediment for another 15 min. Before inoculation of the MGIT PZA medium culture tubes (pH 5.9), the bacterial suspension was adjusted to a McFarland turbidity of 0.5 and diluted in PBS per the manufacturer’s PZA test protocol.

**Supplementary Figures**

**Supplemental Figure 1**

**
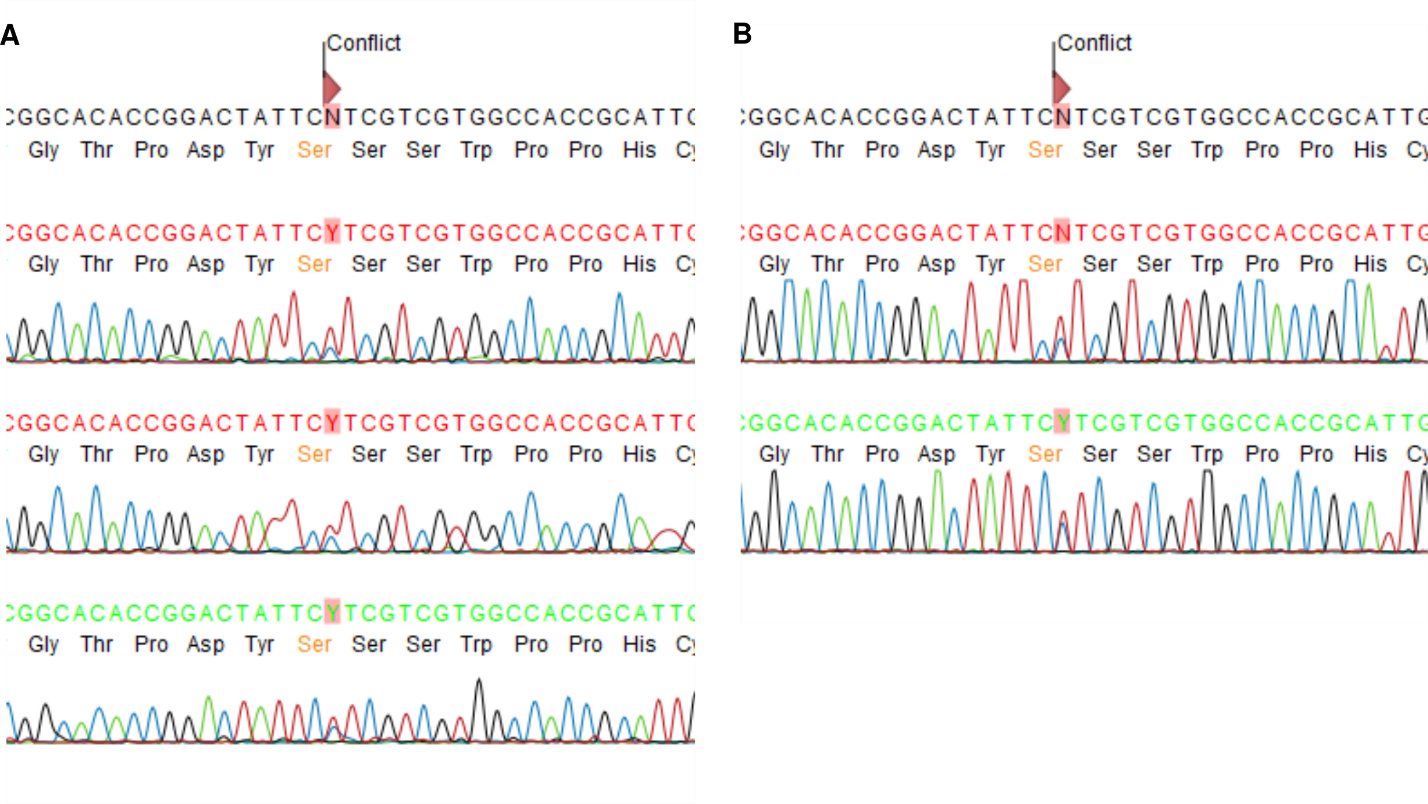
**

**Supplementary Figure Legends**

**Supplementary Figure 1.** **Sanger sequencing from DNA extracted from initial culture.** Sanger chromatograms from (**A**) the original run, around codon 65, where the disputed Ser65Ser synonymous mutation was detected, and **(B)** Replication of Sanger sequencing on the original DNA extraction by a third-party (Genoscreen).
