# Supplementary figures and images for: Pyrazinamide-resistant Tuberculosis Obscured From Common Targeted Molecular Diagnostics
Source: Drug Resist Updat. Author manuscript; Available in PMC 2023 Jul 3. (PMC10317212; doi:10.1016/j.drup.2023.100959)

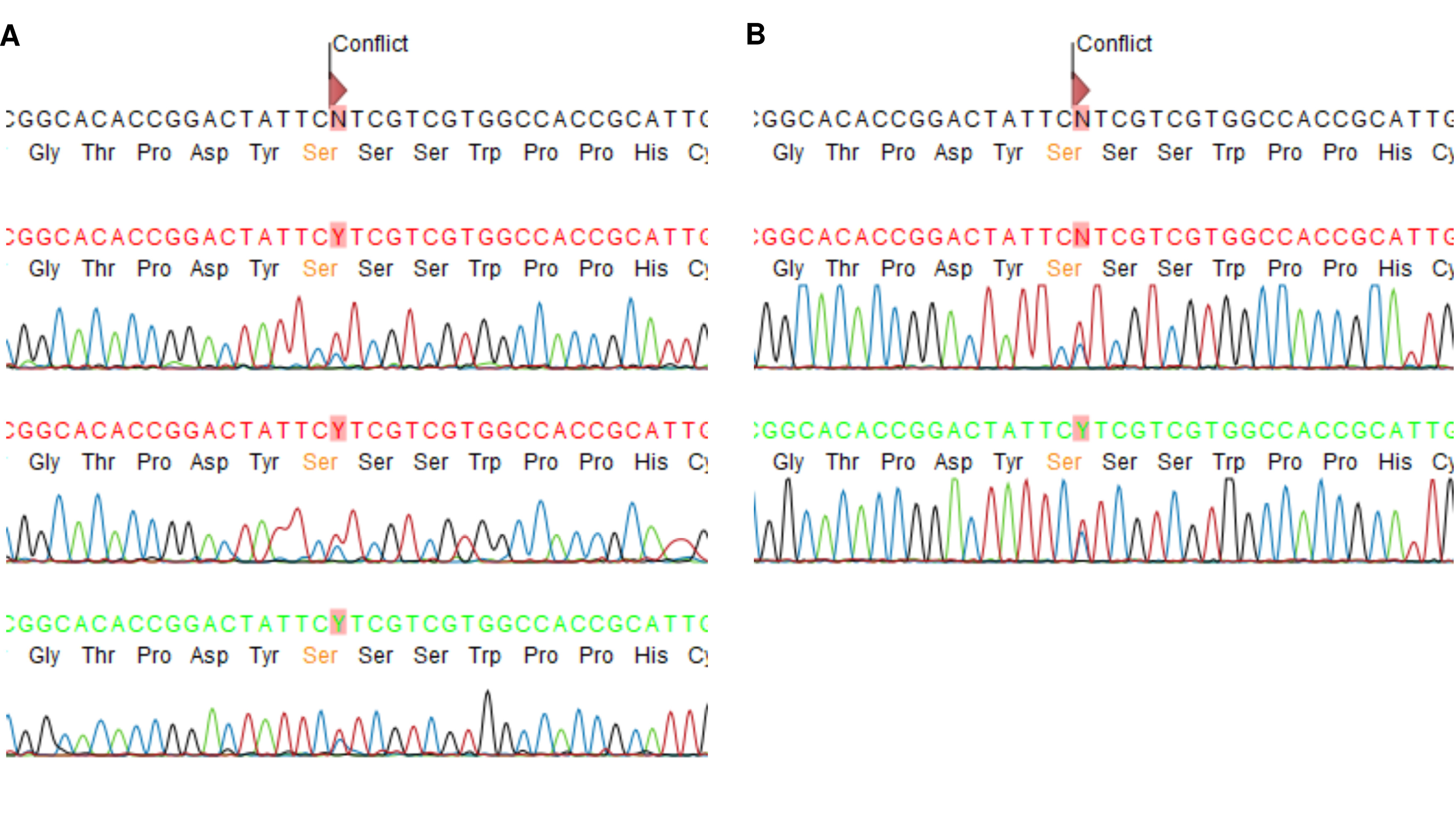

Supplement: Supplementary Material [file NIHMS1907051-supplement-Supplementary_Material.zip › 1-s2.0-S1368764623000420-mmc2_lrg.jpg]

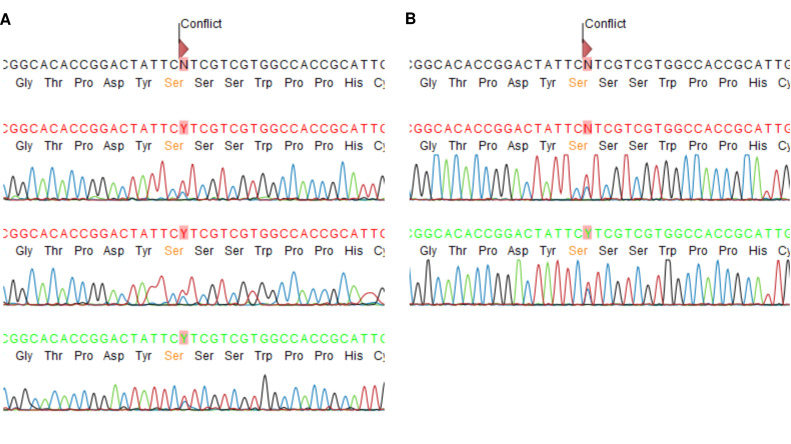

Supplement: Supplementary Material [file NIHMS1907051-supplement-Supplementary_Material.zip › 1-s2.0-S1368764623000420-mmc2.jpg]
